# Euphol from *Euphorbia tirucalli* Negatively Modulates TGF-β Responsiveness via TGF-β Receptor Segregation inside Membrane Rafts

**DOI:** 10.1371/journal.pone.0140249

**Published:** 2015-10-08

**Authors:** Chun-Lin Chen, Ying-Pin Chen, Ming-Wei Lin, Yaw-Bin Huang, Fang-Rong Chang, Tsai-Hui Duh, Deng-Chyang Wu, Wei-Chiang Wu, Yu-Chen Kao, Pei-Hua Yang

**Affiliations:** 1 Department of Biological Sciences, National Sun Yat-sen University, Kaohsiung, Taiwan, ROC; 2 Doctoral Degree Program in Marine Biotechnology, National Sun Yat-sen University and Academia Sinica, Kaohsiung, Taiwan, ROC; 3 Department of Pharmacy, School of Pharmacy, Kaohsiung Medical University, Kaohsiung, Taiwan, ROC; 4 Graduate Institute of Nature Products, College of Pharmacy, Kaohsiung Medical University, Kaohsiung, Taiwan, ROC; 5 Department of Medicinal and Applied Chemistry, Kaohsiung Medical University, Kaohsiung, Taiwan, ROC; 6 Division of Gastroenterology, Department of Internal Medicine, Kaohsiung Medical University Hospital, Kaohsiung, Taiwan, ROC; 7 Faculty of Pharmacy, Kaohsiung Medical University, Kaohsiung, Taiwan, ROC; 8 Center for Stem Cell Research, Kaohsiung Medical University, Kaohsiung, Taiwan, ROC; University of British Columbia, CANADA

## Abstract

Transforming growth factor-β (TGF-β) responsiveness in cultured cells can be modulated by TGF-β partitioning between lipid raft/caveolae- and clathrin-mediated endocytosis pathways. Lipid rafts are plasma membrane microdomains with an important role in cell survival signaling, and cholesterol is necessary for the lipid rafts’ structure and function. Euphol is a euphane-type triterpene alcohol that is structurally similar to cholesterol and has a wide range of pharmacological properties, including anti-inflammatory and anti-cancer effects. In the present study, euphol suppressed TGF-β signaling by inducing TGF-β receptor movement into lipid-raft microdomains and degrading TGF-β receptors.

## Introduction

Transforming growth factor-β is a family of 25-kDa disulfide-linked dimeric proteins. Mammals possess 3 TGF-β isoforms (TGF-β_1_, TGF-β_2_, and TGF-β_3_) which share approximately 70% sequence homology [1, 2]. TGF-β exhibits bifunctional growth regulation: it inhibits the growth of most cell types, including epithelial cells, endothelial cells and lymphocytes; and it stimulates proliferation of mesenchymal cells such as fibroblasts [1, 2]. In epithelial cells, TGF-β inhibits cell proliferation, induces apoptosis, and mediates differentiation, suggesting that TGF-β signaling has a tumor suppressing effect in epithelial tumors [3, 4]. However, TGF-β promotes invasion and metastasis in late stage tumors, indicating its effect on human cancers depends on the stage of the cancer. In addition to growth regulation, TGF-β regulates the synthesis of the extracellular matrix, chemotaxis, angiogenesis, and differentiation of several cell lineages. TGF-β signaling has been implicated in many pathophysiological processes, including wound repair, tissue fibrosis, immunosuppression, and morphogenesis [5]. The primary biological activities of TGF-β are mediated by specific cell surface receptors known as type I and type II TGF-β receptors (TβR-I and TβR-II) [6]. TGF-β exerts its effects on cells by binding to TβR-II, leading to recruitment of TβR-I and subsequent activation of the receptor complex. Smad2 and Smad3 are direct substrates of the activated TGF-β receptor complex. After stimulation, the Smad complex translocates into the nucleus, where it functions as a member of transcription factor complexes that regulate the expression of a variety of genes [2, 3, 7].

**Fig 1:**
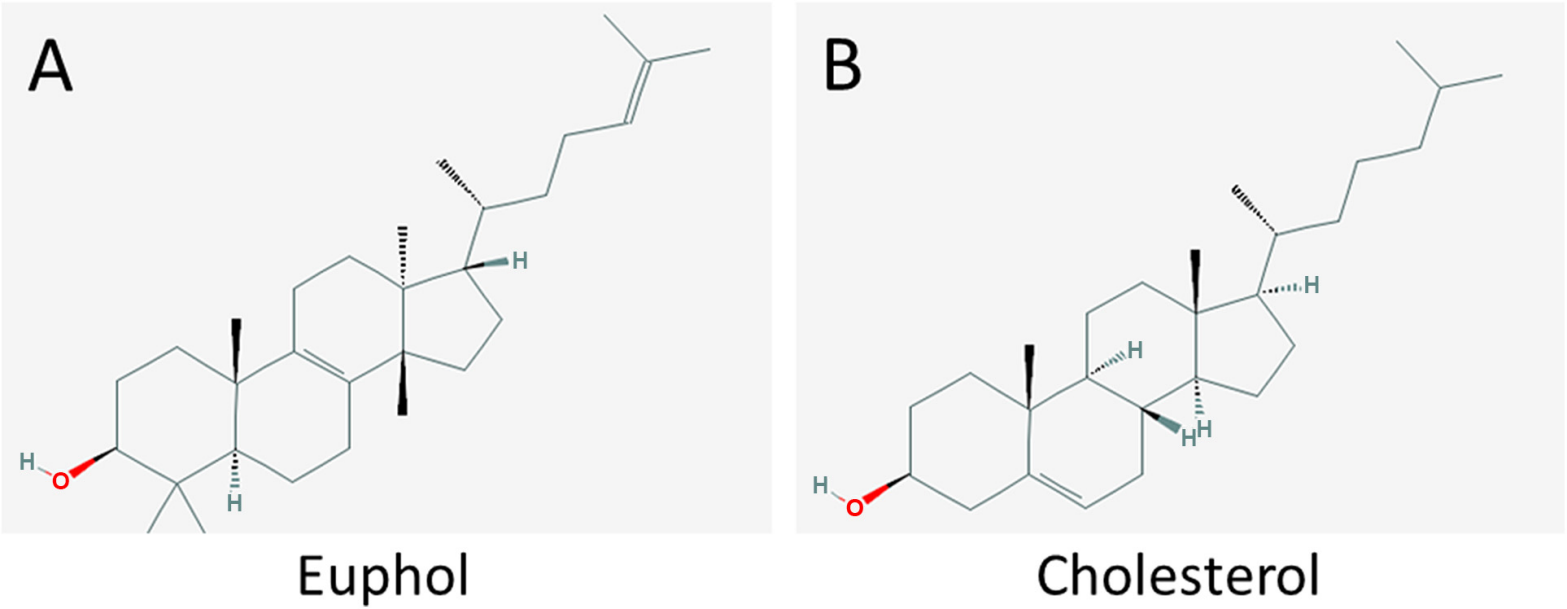
Chemical structures of (A) cholesterol and (B) euphol.

The tetracyclic triterpene euphol (Fig 1A), the main constituent of the sap of *Euphorbia tirucalli* (family *Euphorbiaceae*), is used in folk medicine to treat several types of cancer, including basal cell carcinomas, leukemia, and lung, prostate, and breast cancers [8–13]. Euphol in the ethanolic extract of *E*. *tirucalli* exhibits diverse biological activities, such as anti-viral, anti-inflammatory, and anti-cancer effects [14, 15]. It exerts antiviral effects by inhibiting reverse transcriptase in purified human immunodeficiency virus type 1 [16]. It produces anti-inflammatory effects by mediating nuclear factor kappa-light-chain-enhancer of activated B cells (NF-κB) [17], down-regulation of tumor necrosis factor-α and cyclooxygenase-2 [18], and reduced activation of protein kinase C [19]. According to Wang *et al*., euphol suppresses breast cancer growth by modulating cyclin D1, p21, and p27 expression [20]. It also selectively induces apoptosis in gastric cancer cells by modulating ERK signaling [21]. However, the mechanisms underlying the antitumor effect of euphol remain to be elucidated.

We previously reported that a high concentration of cholesterol in the culture medium suppresses TGF-β responsiveness in cultured cells by causing accumulation of cell-surface TGF-β-TGF-β receptor complexes in lipid rafts/caveolae of the plasma membrane, facilitating rapid degradation of these complexes, and thus attenuating TGF-β-stimulated signaling and related responses [22–24]. It is believed cholesterol mediates this effect by increasing formation or stabilization of lipid rafts/caveolae, presumably via direct integration of cholesterol into the plasma membranes of target cells [22, 23]. Lipid rafts/caveolae are thought to form platforms for protein complexes involved in many key cellular functions, including signal transduction, membrane fusion, organization of the cytoskeleton, lipid sorting, protein trafficking, and localization and activity of specific membrane channels [25–28].

Several studies have shown that the biochemical properties of lipid rafts can be altered by sequestering cholesterol. For example, methyl-β-cyclodextrin (MβCD) removes cholesterol from the plasma membrane and reduces lipid raft integrity, thus modulating lipid raft-dependent cellular events [26, 29, 30]. However, nystatin sequesters cholesterol in cell membranes when present in sufficient concentrations. It forms pores in the membrane and disrupts lipid-rafts [31]. Natural cholesterol derivatives, including steroid hormones and phytosterols also change the structure of lipid rafts, depending on their structural moiety [32–34]. Because euphol (Fig 1B) is structurally similar to cholesterol (Fig 1A), we hypothesized that euphol would modulate TGF-β responsiveness by changing the partitioning of TGF-β receptors between lipid-raft/caveolae and non-lipid raft microdomains on the plasma membrane.

In the present study, euphol induced segregation of TGF-β receptors to lipid rafts, decreased TGF-β-induced phosphorylation of Smad2, and suppressed TGF-β-induced transcriptional activation in Mv1Lu cells. Our data provides insight into the anticancer effects of euphol in gastric cancer cells.

## Methods and Materials

### Materials

Dulbecco's modified Eagle's medium (DMEM), phenylmethanesulfonyl fluoride (PMSF), bovine serum albumin (BSA), and peroxidase-conjugated anti-rabbit IgG were obtained from Sigma (St. Louis, MO). The pre-stained protein ladder (64, 49, 37, 26, and 20 kDa) and fetal calf serum (FCS) was obtained from Invitrogen (Carlsbad, CA). TGF-β was purchased from Austral Biologicals (San Ramon, CA). The polyclonal antibodies against early endosome antigen 1 (EEA1), transferrin receptor (TfR), Smad2, fibronectin, lamin B, EGF receptor, flotillin-1, flotillin-2, CD-55, caveolin-1, TβR-I, and TβR-II were obtained from Santa Cruz (Santa Cruz, CA). The rabbit polyclonal antibody to phospho-Smad2 was purchase from Cell Signaling (Boston, MA).

### Isolation of euphol

The fresh aerial parts of E. *tirucalli* [17] were collected in the experimental greenhouse in Kaohsiung Medical University with appropriate growing conditions, Kaohsiung, Taiwan. Euphol was purified and provided by Fang-Rong Chang who is one of our authors in Kaohsiung Medical University, Kaohsiung, Taiwan. E. *tirucalli* is not endangered or protected species and Dr. Fang-Rong Chang is fully authorized to produce and provide euphol. The latex of the fresh plant was collected drop by drop, and the remaining aerial parts of the plant (15.0 kg) were extracted with MeOH. The evaporated latex MeOH extract (5.9 g) was separated by column chromatography on a silica gel (300 g) with a gradient system of n-hexane/CHCl_3_ (3:1, 2:1, and 1:1, at 800 mL each) and CHCl_3_ (1000 mL), yielding 20 fractions. Fractions of 7–9 (4.6 g) were combined and further purified by a silica gel column (200 g) with n-hexane/CHCl_3_ (3:1, 1500 mL), yielding euphol (4.2 g) as the major constituent triterpene.

### Cell Culture

Mv1Lu cell line (mink lung epithelial cells, CCL-64) and AGS cells (human gastric cancer cell, CRL-1739) were obtained from the American Type Culture Collection (ATCC, Rockville, MD, USA). Mv1Lu cell stably express PAI-1 luciferase promoter was kindly provided by Dr. Jung San Huang (Department of Biochemistry & Molecular Biology, Saint Louis University, Saint Louis, MO, USA). MKN45 (a poorly differentiated human gastric adenocarcinoma) cells were obtained from the Health Science Research Resources Bank (HSRRB, Osaka, Japan). Mv1Lu cells and the gastric cancer cell lines AGS and MKN45 cells were cultured in DMEM supplemented with 10% FCS, 1% penicillin and streptomycin, pH 7.4. The cells were seeded in tissue culture plates (Falcon, Bedford, MA, USA) and incubated at 37°C in a humidified atmosphere of 5% CO_2_. Mv1Lu cells were subcultured twice a week by trypsinization in a 0.25% trypsin-EDTA solution after washing with Ca^2+^- Mg^2+^-free saline.

### Treatment of Mv1Lu cells with TGF-β after preincubation with Euphol

To study the effects of euphol in TGF-β receptor level and responsiveness, cells were grown in 12-well plastic plates (4 × 10^5^ cells/ml with 1 ml/well 10% FCS-DMEM) for 24 hours in a humidified CO_2_ incubator at 37°C. Then, the medium was replaced with fresh 0.1% FCS-DMEM. To observe the TGF-β receptor and fibronectin levels, cells were preincubated with euphol for 1 hour at 37°C. After that, TGF-β was added to the medium and continued incubation for 48 hours; the cultures were then washed twice with cold PBS, and harvested for Western blot analysis. To observe the Smad2 phosphorylation, the cells in 12-well plastic plates were preincubated with euphol for 1 hours in DMEM with 0.1% FCS at 37°C. After that, TGF-β was added to the medium and continued incubation for 30 min.

### Western blot analysis

Cell lysates of Mv1Lu cells (~50 μg protein) were subjected to 10% SDS-PAGE under reducing conditions and then electro-transferred to PVDF membranes. After being incubated with 5% nonfat milk in Tris-buffered saline plus Tween 20 (TBST) (50 mM Tris-HCl, pH 8.0, 150 mM NaCl, 0.05% Tween 20) for 1 hour at room temperature, the membranes were further incubated with specific polyclonal antibodies to TβR-I and TβR-II in TBST/non-fat milk at 4°C for 18 hours and washed three times with TBST for 10 min each. Bound antibodies were detected using peroxidase-conjugated anti-rabbit IgG and visualized using the ECL system (ImageQuant).

### Luciferase activity assay

Mv1Lu cells were transiently transfected with fibronectin [35] and collagen [36] luciferase promoter plasmids using electroporation. The cell suspension was mixed with 14 μg/ml reporter plasmid DNA and 0.1 μg/ml β-galactosidase plasmid then transferred into an electroporation cuvette (0.4 cm gap, Bio-Rad, Hercules, CA) and pulsed (950 μF, 250 V, Gene Pulser II, Bio-Rad). After electroporation, cells were seeded in 24-well cluster plates (Corning) and continued grown for 24 hours. In other experiments, Mv1Lu cells stably expressing the luciferase reporter gene driven by the PAI-1 promoter (MLECs-Clone 32) grown to near confluence on 24-well plates were treated with different concentrations of euphol, with and without 25 μg/ml nystatin or 0.5% MβCD at 37°C for 1 hour, respectively. Treated cells were further incubated with 50 pM TGF-β at 37°C for 4 hours and lysed in 100 μl of lyses buffer (Promega). The cell lysates (~20 μg protein) were then mixed with D-luciferin (Gold Biotechnology, St. Louis, MO, U.S.A.) assay buffer and assayed using the luminometer (Titertek-Berthold, Pforzheim, Germany). β-galactosidase activity in the lysates was determined using β-galactosidase substrate solution (0.2 M H_2_PO_4_, 2 mM MgCl_2_, 4 mM ONPG, 0.25% β-mercaptoethanol) and spectrophotometry. The luciferase activity was normalized for β-galactosidase activity and the relative increase in corrected luciferase activity was calculated versus controls.

### Immunofluorescent detection of Smad2

Cells were grown on 24 mm round coverslips (Paul Marienfeld). After 2 hours of serum starvation and pretreatment with or without 5 μg/ml euphol, cells were stimulated with TGF-β(20 pM) for 30 min. Cells were washed with phosphate buffered saline (PBS) and fixed in cold methanol for 10 min. After washings with PBS, cells were blocked with 5% goat serum (Dako) in 1% BSA/PBS. After incubation with mouse-anti-Smad2 (H-2; Santa Cruz Biotechnology) at 1:100 dilution in 1% BSA/PBS for 18 hours at 4°C, cells were incubated with donkey anti-mouse FITC (Gene Tex) at RT for 1 h. Coverslips were mounted with slow fade gold anti-fade reagent and DAPI (Invitrogen). Photomicrographs were taken with a Zeiss Axio Observer Z1 microscope equipped with a Photometrics HQ2 camera.

### Immunofluorescent confocal microscopy

Mv1Lu cells transiently expressing TβR-II-HA [37] were grown on coverslips overnight (50% confluency) and pretreated with 5 μg/ml euphol at 37°C for 1 hour and then stimulated with 100 pM TGF-β1 for 30 minutes. After TGF-β stimulation, cells were fixed in methanol at -20°C for 15 minutes, washed with PBS and then blocked by 0.2% gelatin in PBS for 1 hour. Cells were incubated overnight at 4°C in a humidified chamber with a mouse antibody against hemagglutinin (HA) protein (F-7; Santa Cruz Biotechnology) and rabbit antibody against caveolin-1 (N-20; Santa Cruz Biotechnology) at 1:100 dilution. After extensive washing, cells were incubated with Rhodamine-conjugated donkey anti-mouse antibody and FITC-conjugated goat anti-rabbit antibody at a 1:50 dilution for 1 hour. Images were acquired using a Leica TCS SP confocal microscope (Leica Microsystems Ltd., Heidelberg, Germany). The measurements of colocalization rate were analyzed using a Leica Application Suite.

### Separation of lipid raft and non-lipid raft microdomains of plasma membranes by sucrose density gradient ultracentrifugation

Sucrose density gradient analysis was performed at 4°C as described previously [38]. Briefly, cells were grown to near confluence in 100 mm dishes (5–10×10^6^ cells per dish). Cells were incubated with euphol (5 μg/ml) with or without nystatin (25 μg/ml) at 37°C for 4 hour. After two washes with ice-cold PBS, cells were scraped into 0.85 ml of 500 mM sodium carbonate, pH 11.0. Homogenization was carried out with 10 strokes of a tightfitting Dounce homogenizer followed by three 15-second bursts of an ultrasonic disintegrator (Qsonica, Newtown, CT, USA) to disrupt cell membranes. The homogenates were adjusted to 45% sucrose by addition of 0.85 ml of 90% sucrose in 25 mM 2-(*N*-morpholino) ethanesulfonic acid, 0.15 M NaCl (MBS), pH 6.5, and placed at the bottom of an ultracentrifuge tube. A discontinuous sucrose gradient was generated by overlaying 1.7 ml of 35% sucrose and 1.7 ml of 5% sucrose in MBS on the top of the 45% sucrose solution and it were then centrifuged at 40,000 rpm for 16–20 hours in a P50S2 rotor (Himac, Tokyo, Japan). Ten 0.5-ml fractions were collected from the top of the tube, and a portion of each fraction was analyzed by SDS-PAGE followed by Western blot analysis using antibodies to TβRI (ALK-5), TβRII, TfR-1, EEA-1, and caveolin-1. The relative amounts of TβRI, TβRII, TfR-1 and caveolin-1 on the blot were quantified by densitometry. The total protein recovery and caveolin-1 and TfR-1 localization (fractions 4 to 5, and 8 to 10, respectively) did not significantly changed with any of the treatment protocols.

### RNA isolation and RT-PCR

AGS cells were treated with TGF-β (100 pM) in 0.1% BSA in the presence or absence of euphol and total RNA was isolated using TRIzol reagent (Invitrogen) according to the manufacturer’s instructions. cDNA were synthesized using M-MLV Reverse Transcriptase (Promega, Madison, WI, USA) and oligo dT primer over 1 hour at 42°C. The synthesized cDNA was subjected to PCR amplification using Taq polymerase (Promega) and the following gene-specific primers: GAPDH, (forward) 5-GCATGGCCTTCCGTGTTC-3 and (reverse) 5-GATGTCATCATACTTGGCAGGTTT-3; TβR-I, (forward) 5-CGTGCTGACATCTATGCAAT-3 and (reverse) 5-AGCTGCTCCATTGGCATAC-3; and TβR-II, (forward) 5-TGCACATCGTCCTGTGGAC-3 and (reverse) 5-GTCTCAAACTGCTCTGAAGTGTTC-3 and fibronectin, (forward) 5-CTGGGATGCTCCTGCTGTCAC-3 and (reverse) 5-CTGTTTGATCTGGACCTGCAG-3. Amplified DNA was analyzed by agarose gel electrophoresis.

### Cytotoxicity assay

The cytotoxic effect of euphol was determined by incubating cells at a density of 1×10^3^ per well of 96-well flat bottomed plates (Costar) 24 hours before treatment. Cells were incubated with 100 μl/well of DMEM containing 0.1% FCS and increasing concentrations of euphol (range 0–60 μg/ml) for 24 hours. After incubation, cells were washed twice with PBS. Next, 50 μl of 1 mg/ml 3-(4,5-dimethylthiazol-2-yl)-2,5-diphenyltetrazolium bromide (MTT) (Sigma Co.) solution was added to each well, followed by a 2 hours incubation at 37°C. The formazan crystals were dissolved using 150 μL of DMSO and absorbance was measured at 595 nm on a Spectra Fluor multiwell plate reader (Tecan). Results are presented as percentage of survival taking the control (untreated cells) as 100% survival.

### Preparation of lipid vesicles for SPR

The lipid vesicles for SPR analysis were prepared base on previous report with modification [39]. Small unilamellar lipid vesicles prepared by hydrating the required amount of dried lipid with the desired buffer; 10mM Hepes at pH 7.0 buffers were deoxygenated with nitrogen gas and the hydrated lipids were maintained under a nitrogen atmosphere. Phospholipids in chloroform solutions were dried under a rotary evaporator and the resulting lipid film was left under vacuum overnight to remove all traces of organic solvent. After lipid hydration the resulting multilamellar liposome suspension was sonicated in a bath sonicator until a clear suspension of small unilamellar vesicles was obtained. Before deposition on the SPR chip, lipid suspensions were subjected to several cycles of vortexing and warming at 40°C.

### Surface plasmon resonance (SPR) analysis

The binding parameters for the interaction of euphol and lipid membrane were assayed with the BIAcore 3000 system using the Biacore pioneer chip L1 (BIAcore, Uppsala, Sweden). The gold surface of the L1 chip is coated with a dextran layer containing lipophilic molecules for efficient capture of lipid vesicles onto the surface, resulting in an extended lipid bilayer over the dextran layer. The chip was equilibrated cleaned with 40mM octyl-glucoside (40 μL), and washed thoroughly with 10mM Hepes buffer. Lipid vesicles were deposited on the L1 chip by injecting four 100 μL of their aqueous suspensions containing 0.5 mM lipid, washed twice with 50 mM NaOH to stabilize the deposited lipid layer and followed by a buffer injection to remove alkali. Fresh solutions of euphol in Hepes buffer were injected at various concentrations (0, 25, and 50 μM). The chip surface was regenerated with two pulses of 50 mM NaOH. Binding data were evaluated using the BIAevaluation 4.1 program (BIAcore).

### Mass spectrometry

Euphol analysis was carried out on an Agilent 1200 HPLC system(Agilent Technologies) coupled to an API 4000 triple quadrupole mass spectrometer (Applied Biosystem, Foster City, CA, USA). A Thermo HyPURITY ADVANCE (250x4.6 mm, 5 mm) and a mixed solvent of methanol-ammonium acetate buffer (2mM) (95:5) at a flow-rate 1.0 mL/min were used. The electrospray negative mode was selected as an ion source for detection. The qualitative was performed in multiple reactions monitoring (MRM) mode with the precursor-to-product ion transitions 426 to 96 and 426 to 80. The optimized ESI source parameters were as follows: ion spray voltage, -4500 V; nitrogen nebulizer gas pressure, 45 psi; nitrogen auxiliary gas pressure, 60 psi; nitrogen curtain gas pressure, 15 psi; heater temperature 55°C and collisionally activated dissociation (CAD) gas.

### Statistical analysis

Experiments were repeated at least three times, and the number of repetitions is represented in the figure legends by ‘‘n = “. Data are expressed as the mean ± SEM. Group differences were analyzed with one-way analysis of variance (ANOVA). Statistical significance was considered at P<0.05 (*) or P<0.01 (**).

## Results

### Euphol blocks TGF-β-induced transcriptional responses

**Fig 2:**
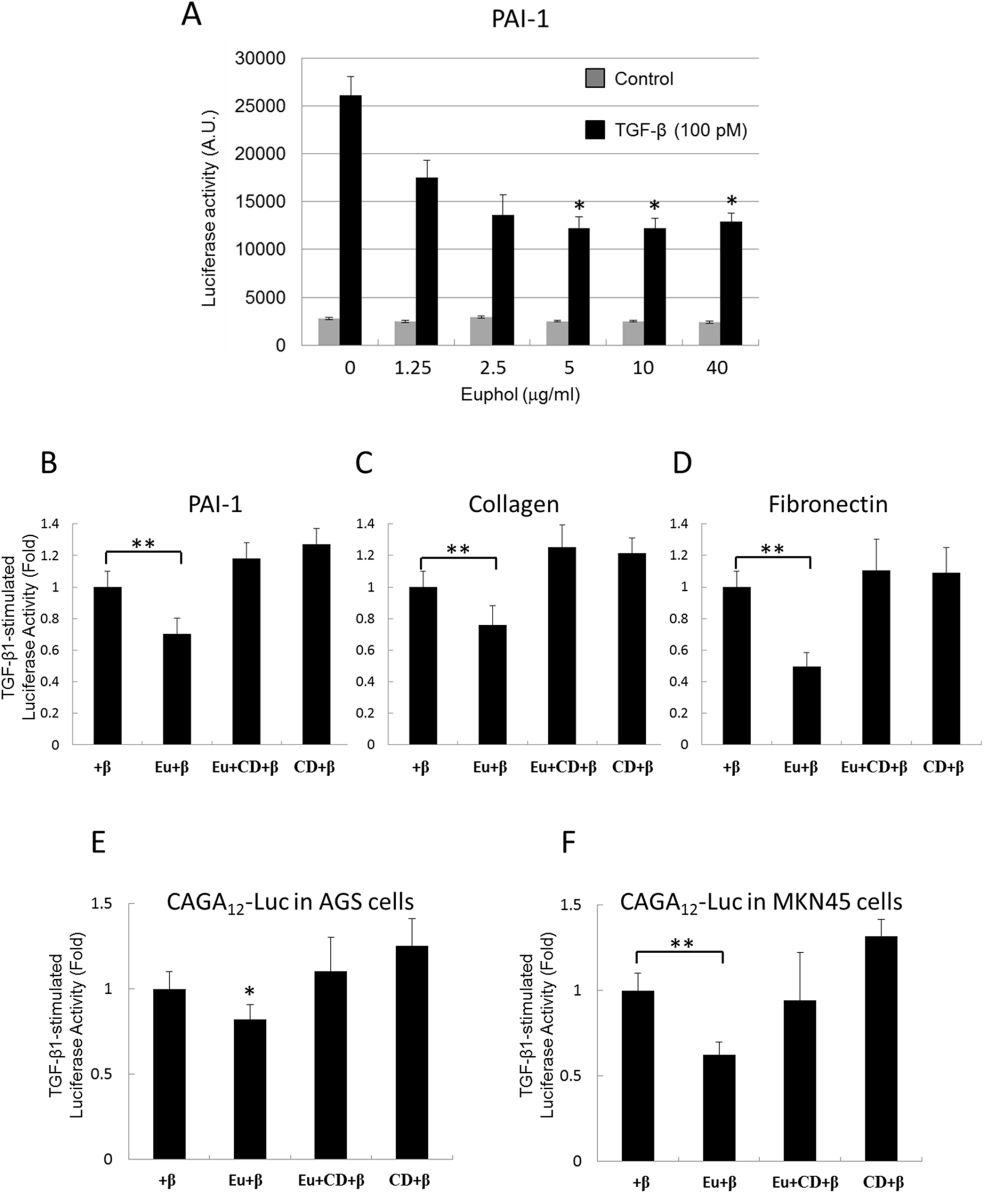
Inhibition of TGF-β-induced transcriptional activation by euphol. (A) Mv1Lu cells with stable expression of the PAI-1 luciferase promoter were treated with increasing concentration of euphol. The gray bars in (A) represent the cells without TGF-β treatment. The black bars represent the cells treated with 100 pM TGF-β. (B,C,D) Mv1Lu cells were transfected with CAGA_12_-Luc, collagen, or a fibronectin luciferase plasmid, and AGS (E) and MKN45 (F) gastric cancer cells were transfected with CAGA_12_-Luc, Mv1Lu, AGS, and MKN45 cells were treated with TGF-β (100 pM, +β), euphol (Eu), or MβCD (CD). Luciferase activity was measured as described in the methods section. Columns show mean of three independent experiments performed in triplicated; bars indicate s.d.; *P<0.05 (compare with TGF-β treatment), **P<0.01.

Mv1Lu cells were used to examine the effect of euphol on TGF-β signaling. The Mv1Lu cell line is a model cell line for the study of TGF-β, because it shows a good dose-dependent response to TGF-β (as determined by assays for cell growth), Smad2 phosphorylation, as well as stable expression of a luciferase reporter gene driven by the PAI-1 promoter [38, 40]. In our study, TGF-β (100 pM) induced luciferase activity about 10-fold in Mv1Lu cells (Fig 2A). Euphol treatment reduced TGF-β-dependent luciferase activity in a concentration-dependent manner. For example, cells treated with 1.25 μg/mL euphol showed a 35% reduction in luciferase activity, and cells treated with 5 μg/ml euphol showed a 50% reduction in luciferase activity (Fig 2A). Euphol also suppressed TGF-β-induced transcriptional activation of collagen and fibronectin as determined by transient transfection with collagen and fibronectin luciferase promoter constructs (Fig 2C and 2D). TGF-β (100 pM) stimulated (CAGA)_12_-Luc, collagen, and fibronectin activities by 12-fold, 8-fold and 10-fold, respectively in Mv1Lu cells. The y-axis of Fig 2B–2F represents the units for fold changes of TGF-β-stimulated luciferase activity.

We hypothesized that euphol suppresses TGF-β responsiveness by inducing lipid-raft segregation of TGF-β receptors in the plasma membrane. To study this, Mv1Lu cells were treated with 0.5% MβCD, a cholesterol chelator that destroys lipid raft microdomains [41]. As shown in Fig 2, MβCD suppressed the inhibitory effect of euphol on TGF-β-mediated transcriptional activation of PAI-1, collagen, and fibronectin (Fig 2B, 2C and 2D, Eu+β versus Eu+CD+β). It was noted that MβCD alone did not change TGF-β-induced transcriptional activation. All treatments of euphol used in this study did not affected cell viability, as determined by measurement of mitochondria activity (S1 Fig).

To determine the effect of euphol on TGF-β-stimulated signaling in gastric cancer cells, we used AGS and MKN45 gastric cancer cell lines that were transiently transfected with the TGF-β-responsive luciferase promoter construct (CAGA)_12_-Luc and CAGA boxes within the promoter region of the human PAI-1 gene confer TGF-β and activin stimulatory activity, but not bone morphogenetic protein (BMP) stimulatory activity, on heterologous promoter reporter constructs [42]. TGF-β (200 pM) induced (CAGA)_12_-Luc activity about 6-fold in AGS cells. Euphol (5 μg/ml) treatment inhibited TGF-β-induced luciferase activity, but treatment with MβCD inhibited euphol’s effect (Fig 2E and 2F).

### Euphol attenuates TGF-β-induced Smad2 phosphorylation and nuclear translocation

**Fig 3:**
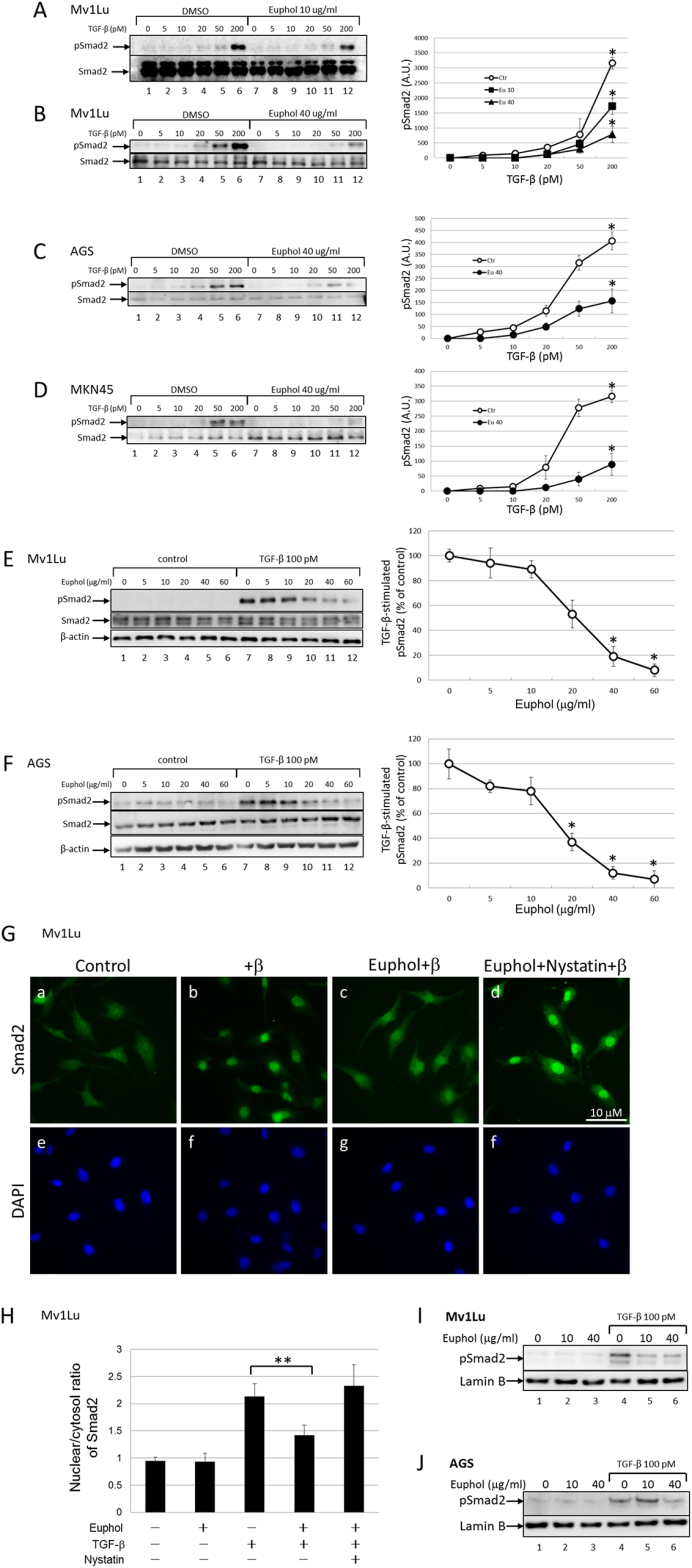
(A) Euphol suppresses TGF-β-induced Smad2/3 phosphorylation. Mv1Lu cells were pretreated with 10 μg/ml euphol for 1 h and stimulated with different concentration of TGF-β for 30 min. (B) Treatment with high concentrations of euphol (40 μg/ml) shows stronger inhibition in Smad2/3 phosphorylation than cells treated with 10 μg/ml euphol (Fig 3A). Results from Fig 3A and 3B were quantified by densitometry showing in upper right panel (C) AGS cells and (D) MKN45 cells pretreated with euphol (40 μg/ml) were stimulated with increasing concentration of TGF-β (0, 5, 10, 20, 50, and 200 pM) and then whole-cell lysates were blotted with antibodies as indicated. (E) Mv1Lu cells and (F) AGS cells pretreated with increasing concentration of euphol (0, 5, 10, 20, 40, and 60 μg/ml) were stimulated with 100 pM and then whole-cell lysates were blotted with the antibodies as indicated. The band intensity was quantitated and the statistical analysis of three independent experiments was provided (*P<0.05). (G) Euphol blocks TGF-β-induced nuclear localization of Samd2/3. TGF-β induces nuclear translocation of Smad2/3 after 30 min (Gb), and this effect is blocked by euphol (Gc). Nystatin reverses euphol induced inhibition of Smad nuclear localization (Gd), the distribution pattern of Smad2/3 was detected by immunofluorescence staining with an anti-Smad2/3 antibody. Bar, 10 μm (Right). The nuclear/cytoplasmic ratio of the Smad2/3 signal was quantified (H). Data are means ± SEM for ≥10 fields. s.d.; **P<0.01 versus TGF-β-induced. (I) Mv1Lu cells and (J) AGS cells treated with euphol were exposed to TGF-β for 45 min before nuclear protein extraction. Nuclear translocation of pSmad2 was observed after SDS-PAGE (10%) followed by Western blot analysis. Lamin B was used to check nuclear isolation and loading.

To examine the effect of euphol on of Smad2 phosphorylation, we performed a western blot analysis to detect phosphorylated Smad2 protein in Mv1Lu, AGS, and MKN45 cells. We pretreated the cells with low (Fig 3A; 10 μg/ml) and high concentrations (Fig 3B, 3C and 3D; 40 μg/ml) of euphol for 1 hour, then stimulated them with increasing concentrations of TGF-β for 30 min. As shown in Fig 3A, TGF-β increased the abundance of phosphorylated Smad2 in cells tested (lane 6), and euphol inhibited this effect (lane 12) when high concentrations of TGF-β were used (Fig 3B, 3C and 3D; lanes 4 through 6 versus lanes 10 through 12). The highest concentration of TGF-β tested was 200 pM (Fig 3A and 3B). Addition of 10 μg/ml and 40 μg/ml of euphol to that system attenuated Smad2 phosphorylation by 40% and 80% respectively (lane 6 versus lane 12).

To further test the concentration dependence of euphol activity, we treated Mv1Lu and AGS cells with increasing concentration of euphol. Fig 3E and 3F illustrate the effects of euphol concentrations on suppression of Smad2 phosphorylation in both Mv1Lu cells and AGS cells. Euphol treatment appreciably inhibited Smad2 phosphorylation at concentrations of 20–60 μg/ml (Fig 3E and 3F, lanes 10 through 12). At 40 μg/ml, euphol suppressed Smad2 phosphorylation by ~81% and ~93% in Mv1Lu cells and AGS cells, respectively. However, mitochondrial activity measurements demonstrate that euphol did not affect cell viability at any concentration (S1 Fig).

In addition, we examined the effect of euphol on translocation of Smad2 to the nucleus, where it activates transcription. Immunofluorescent detection indicated that TGF-β stimulation induced nuclear translocation of Smad2 after 30 min in Mv1Lu cells, and this effect was attenuated by treatment with euphol (Fig 3Gb versus 3Gc). Quantification of the ratio of nuclear Smad2 to cytoplasmic Smad2 in 40 cells from 4 separate experiments revealed that euphol suppressed TGF-β-induced Smad2 nuclear translocation in all of the treated cells (Fig 3H). Furthermore, nystatin, a cholesterol chelator, abolished the inhibitory effect of euphol on TGF-β-induced Smad2 nuclear translocation in 40 ± 5% of the cells (Fig 3H).

To test the inhibitory effect of euphol on pSmad2 nuclear translocation in a more quantitative way, we performed a Western blot to analysis to analyze pSmad2 in the nuclear fraction of euphol-treated cells. The results show a significant increase in the nuclear translocation of pSmad2 within 45 min of TGF-β treatment (Fig 3I and 3J, Lane 1 versus Lane 4), which persisted until the end of the experiment (60 min). It is important to note that euphol treatment significantly suppressed TGF-β-induced pSmad2 phosphorylation and nuclear translocation of pSmad2 (Fig 3I and 3J; lanes 4 versus lane 6). Taken together; these results suggest that euphol treatment suppresses TGF-β-induced signaling.

### Euphol increases accumulation of TGF-β receptors in lipid rafts and caveolae, resulting in enhanced TGF-β-induced degradation

**Fig 4:**
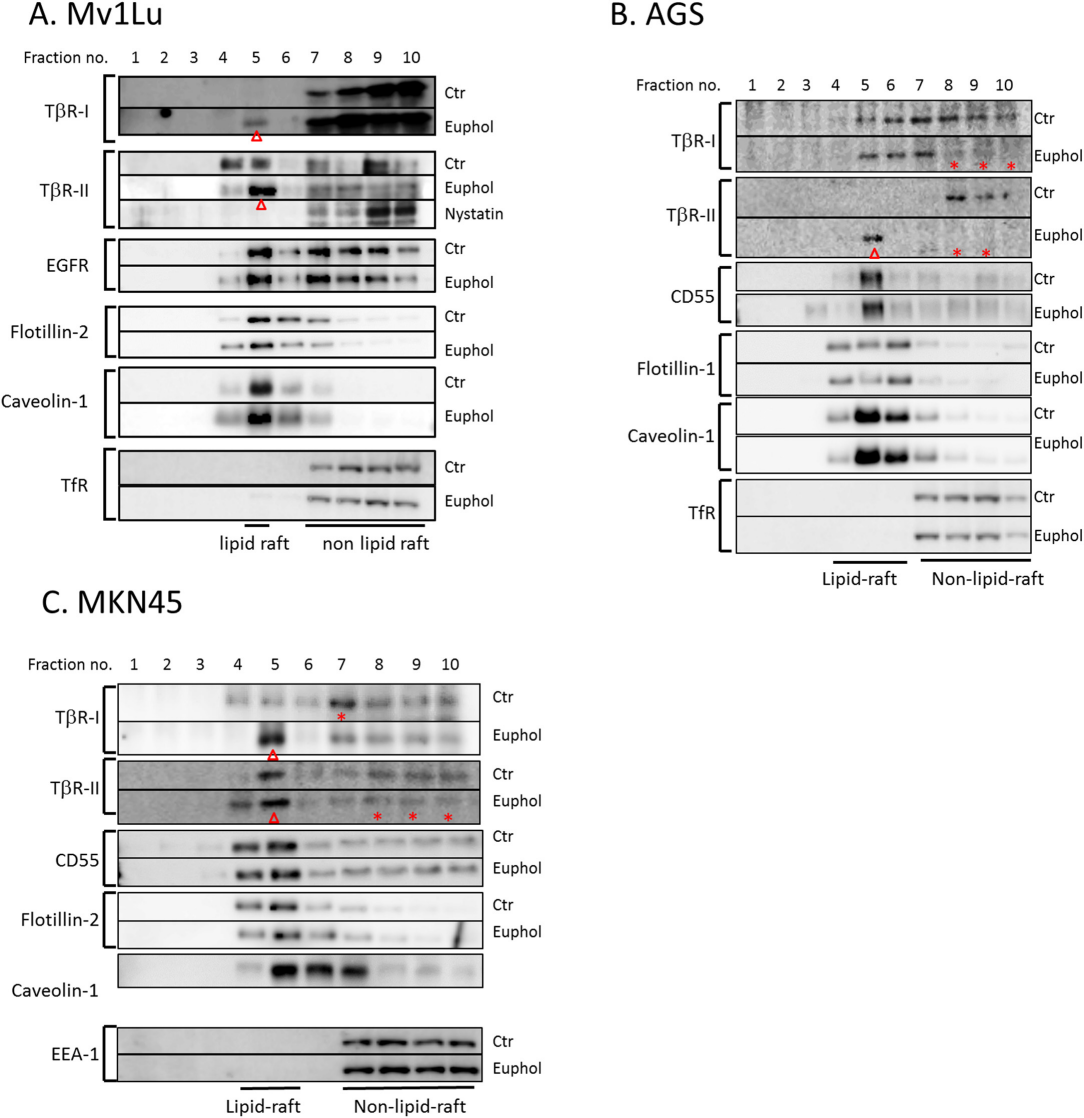
Sucrose density gradient analysis of TGF-β receptors in the plasma membranes of Mv1Lu (A), AGS (B), and MKN45 (C) cells treated with or without euphol and nystatin. Cells were treated with or without 10 μg/ml euphol at 37°C for 1 hour, and the cell lysates from these treated cells were subjected to sucrose density gradient ultracentrifugation. The sucrose gradient fractions were then analyzed by Western blot analysis using anti-TβR-I, anti-TβR-II, anti-TfR-1, anti-EGFR, anti-flotillin, anti-CD-55 and anti-caveolin-1 antibodies. Fractions 4 and 5 contained lipid rafts/caveolae whereas fractions 7–10 were non-lipid raft fractions. Treatment with euphol and nystatin did not affect the abundance of TGF-β receptor proteins and other cell proteins (S2 Fig). Open arrowheads (**ρ**) indicate increased abundance of TβR-I or TβR-II in the fraction in comparison with that of the untreated control cells. The stars (*) indicate decreased abundance of TβR-I or TβR-II in the fraction in comparison with that of the untreated control cells. In Fig 4C, due to the poor expression level of TfR in MKN45 cells, we show the blot of early endosome antigen-1 (EEA-1), which is an alternative marker for non-lipid raft fractions.

We previously demonstrated that TGF-β responsiveness is determined by the localization of TβR-I and TβR-II in lipid rafts and caveolae, as opposed to non-lipid raft microdomains, in plasma membranes [38, 43]. To test the effect of euphol on the partitioning of TGF-β receptors between lipid raft and non-lipid raft microdomains in the plasma membrane, we used sucrose density gradient ultracentrifugation analysis [44] and confocal microscopy to analyze the localization of TβR-I and TβR-II in treated (euphol 10 μg/mL) and untreated cells. As shown in Fig 4A, TβR-I was found primarily in the non-lipid raft fractions (fractions 7 through 10) of Mv1Lu cells. In contrast, TβR-II was present in both the non-lipid raft and lipid raft-caveolae fractions (fractions 4 and 5), which contained transferrin receptor 1 (TfR-1) and caveolin-1, respectively. TfR-1 and caveolin-1 localization were not changed by any of the treatment protocols. Flotillin-1, flotillin-2, and CD55 are also specific markers for lipid raft/caveolae-containing fractions. Euphol treatment neither changed the localization nor altered expression levels of these three markers in the cell lines tested. Lipid raft-caveolae fractions in the plasma membrane of Mv1Lu cells, AGS cells, and MKN45 cells were enriched with TβR-I and TβR-II after treatment with euphol compared with the same fractions before treatment (Fig 4A, 4B and 4C).

Lipid raft localization has been shown to regulate epidermal growth factor receptor (EGFR) signaling [45]. For example, alteration of the lipid raft localization of EGFR modifies receptor dimerization and EGF-stimulated ERK phosphorylation [46]. In this study, euphol did not change membrane partitioning of EGFR between lipid rafts and non-lipid rafts (Fig 4A). This result indicates that euphol specifically changes TGF-β receptor membrane localization and activities.

**Fig 5:**
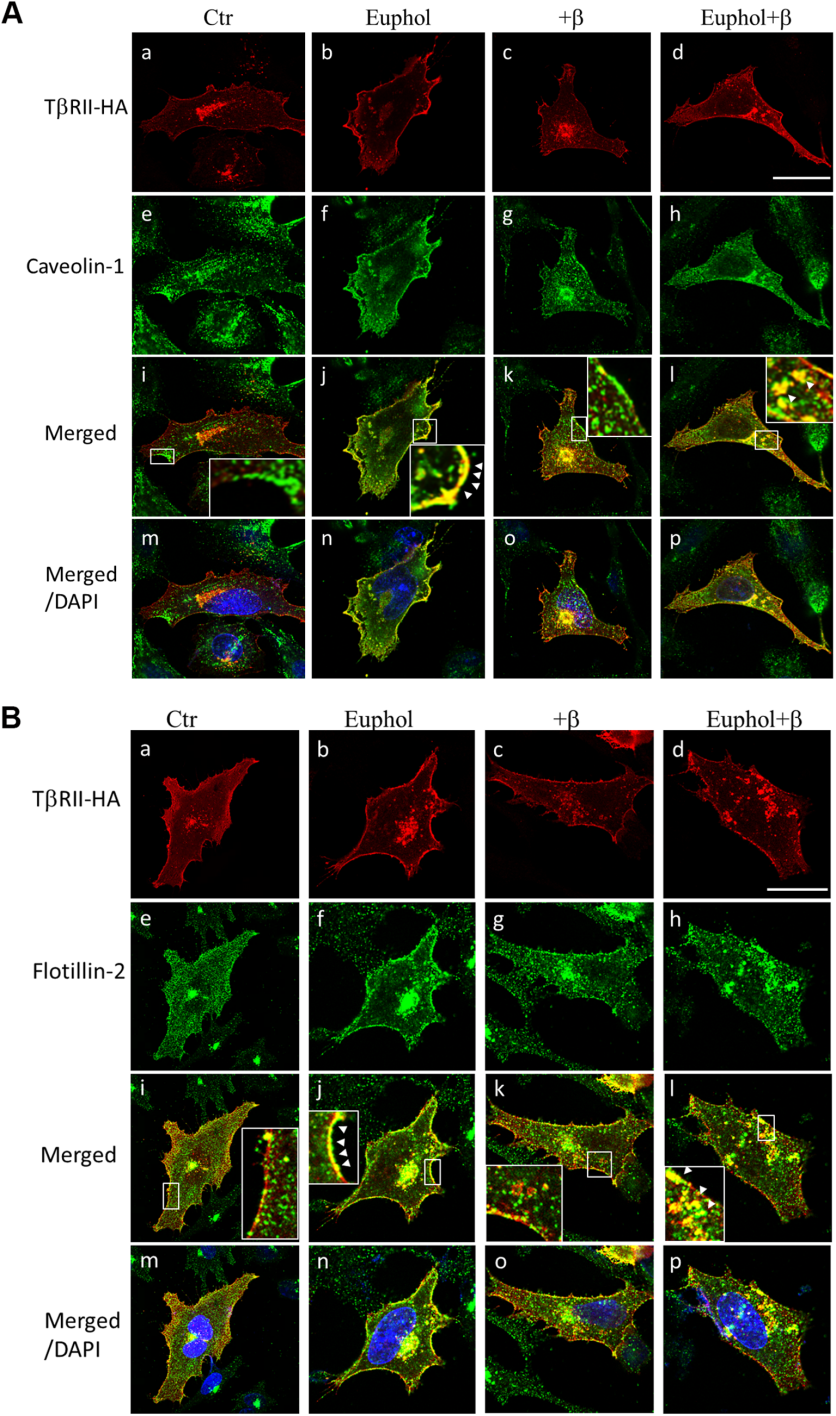
Immunofluorescent localization of TβR-II and caveolin-1(or flotillin-2) in Mv1Lu cells treated with and without euphol and TGF-β. Mv1Lu cells that transiently expressed TβR-II-HA were treated with or without 10 μg/ml euphol at 37°C for 1 hour, after which they were incubated with or without 100 pM TGF-β at 37°C for 30 minutes. The cells were then fixed with cold methanol and incubated with mouse anti-HA antibodies (Fig 5A and 5B, a-d), rabbit anti-caveolin-1 antibodies (Fig 5A, e-h), or rabbit anti-flotillin-2 antibodies (Fig 5B, e-h) followed by incubation with rhodamine-conjugated donkey anti-mouse antibodies or FITC-conjugated goat anti-rabbit antibodies. The fluorescence in the cells was measured using confocal fluorescence microscopy. Bar, 20 μm. The white arrows indicate colocalization of TβR-II-HA and caveolin-1 (or flotillin-2) at the cell surface (j) and in endocytic vesicles (l).

To confirm that fractions 4 and 5 in the sucrose gradient contained lipid raft domains, we treated Mv1Lu cells with nystatin, a lipid raft-disrupting agent [22], prior to solubilization for sucrose gradient fractionation. Nystatin treatment resulted in movement of TβR-II to the high-density fractions (fractions 9 and 10; Fig 4A) and the complete absence of TβR-II from fraction 5. This result suggests that TβR-I and TβR-II were tethered to cholesterol-enriched microdomains after euphol treatment. As shown by immunofluorescence confocal microscopy (Fig 5Aj and 5Bj), treatment with 10 μg/mL euphol for 1 hour caused TβR-II to colocalize with caveolin-1 (Fig 5A) and flotillin-1 (Fig 5B) at the cell surface. Treatment with both euphol and TGF-β resulted in localization of TβR-II in caveolin-1 and flotillin-1 enriched vesicles (Fig 5Al and 5Bl). The Cav1- and TβR-II-positive spots may be endocytic caveolar carrier/endocytic vesicles.

### Euphol treatment resulting in enhanced TGF-β receptor degradation in Mv1Lu and MKN45 cells

**Fig 6:**
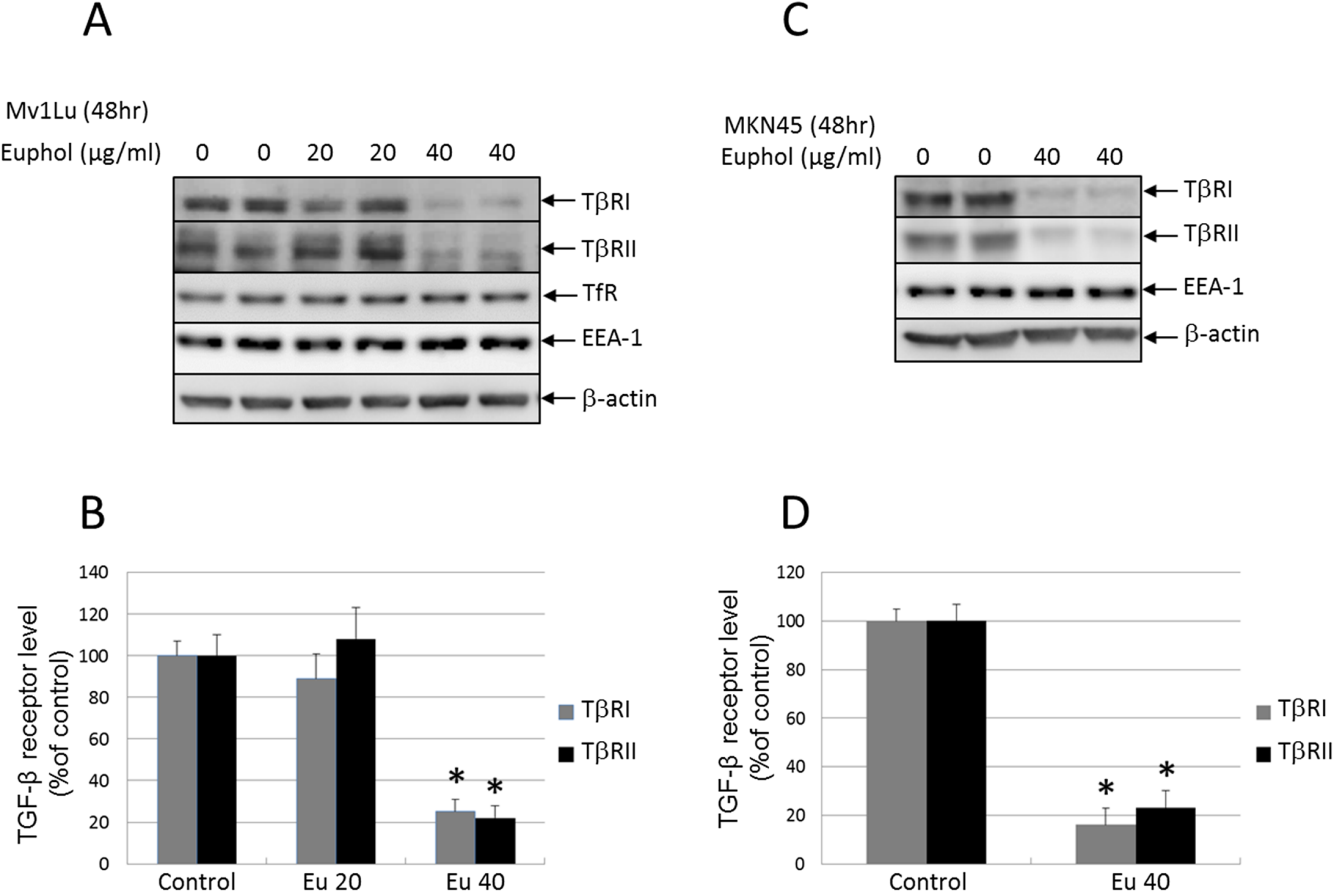
Euphol decreased the abundance of TβR-I and TβR-II in Mv1Lu (A) and MKN45 (B) cells after. Mv1Lu and MKN45 cells were treated with several concentrations of euphol at 37°C for 48 hours, after which the cell lysates were subjected to Western blot analysis using anti-TβR-I, anti-TβR-II, TfR, EEA-1, caveolin-1, and anti-β-actin antibodies (A and C), followed by quantification by densitometry (B and D). The ratio of the relative amounts of TβR-I, TβR-II, and β-actin in cells treated without euphol was taken as 100% TGF-β receptor expression. The data are representative of a total of four independent analyses; values are mean ± s.d. significantly lower than control cells: *P<0.05 versus control.

In Mv1Lu and MKN45 cells, a short period (1 hr) of euphol treatment increased lipid raft/caveolae accumulation of TβR-I and TβR-II (Fig 4B and 4C). Long-term euphol treatment further facilitated degradation of these receptors as demonstrated by the western blot (Fig 6; 48 hours). These results suggest that euphol treatment enhances TGF-β-induced and lipid raft/caveolae-mediated internalization and degradation of TGF-β receptors. For example, in Mv1Lu cells, euphol (40 μg/ml) increased degradation of TβR-I and TβR-II by ~75% and ~79% respectively (Fig 6A and 6B). In MKN45 cells, euphol (40 μg/ml) enhanced degradation of TβR-I and TβR-II by ~83% and ~75% respectively (Fig 6C and 6D).

To determine whether down-regulation of TGF-β receptors occurs through an increased rate of receptor degradation or decreased rate of receptor synthesis, we used RT-PCR to measure the expression of TβR-I and TβR-II in AGS cells. As shown by the supplemental data (S3 Fig), 24 hours treatment of euphol had no significant effect on the levels of mRNA transcript of TβR-I and TβR-II. This result indicates that euphol accelerates receptor degradation.

### Euphol suppresses TGF-β-induced fibronectin expression

**Fig 7:**
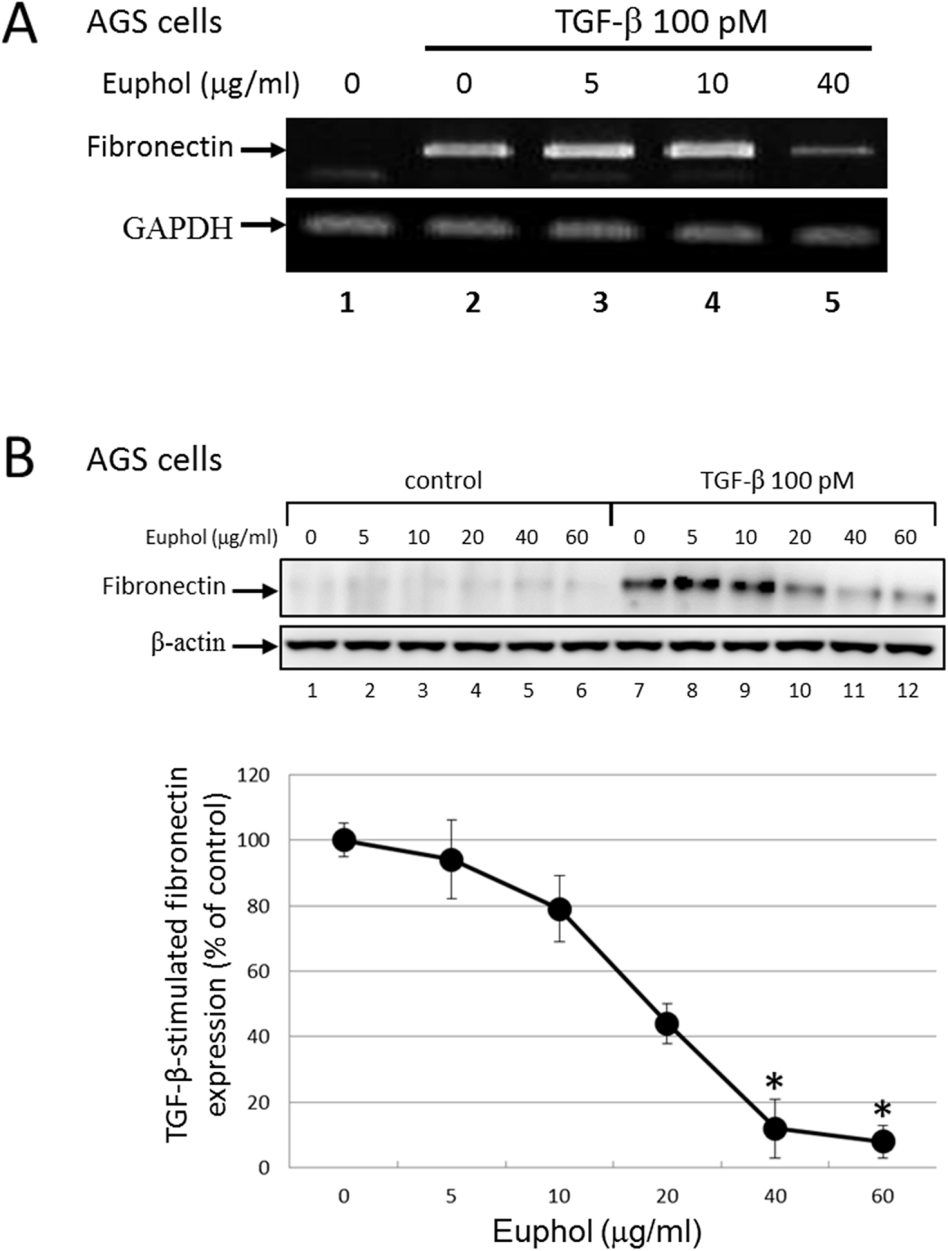
Euphol inhibits TGF-β-induced fibronectin expression. (A) AGS cells were treated with TGF-β (100 pM) ± euphol (5–40μg/ml) for 24 hours. Total RNA were isolated and the expressions of fibronectin were determined by RT-PCR. GAPDH was used as a loading control. (B) AGS cells were treated with TGF-β (100 pM) ± euphol for 48 hours. Total protein extracts from treated cells were Western blotted with anti-fibronectin or anti-β-actin monoclonal antibody. β-actin was used as a loading control; results were quantified by densitometry showing in lower panel. Data represent the means ± s.d. of three independent experiments *P<0.01 versus TGF-β-induced expression.

One important biological activity of TGF-β is transcriptional activation of genes that code for extracellular matrix (ECM) proteins, which are crucial for wound healing, tissue repair, and cancer progression in adult tissues [47, 48]. During prolonged treatment, TGF-β successively induces epithelial-mesenchymal transition which increases expression of ECM proteins such as fibronectin in epithelial cells [47, 49]. This transcriptional activation is mediated by the Smad2 signaling pathway [50, 51]. To determine the effect of euphol on TGF-β responsiveness, we determined the expression of fibronectin mRNA in AGS cells using RT-PCR (Fig 7A). The levels of fibronectin mRNA significantly increased after 24 hr of exposure to TGF-β. However, euphol (40 μg/ml) pretreatment dramatically inhibited TGF-β-stimulated fibronectin expression (Fig 7A; lane 2 versus lane 5).

We also studied the effects of euphol on protein expression in cells using Western blot analysis. In Fig 7B, AGS cells were pretreated with increasing concentrations of euphol at 37°C for 1 hour and then incubated with 100 pM TGF-β at 37°C for 48 hours. Western blot analysis of fibronectin and β-actin proteins in the cell lysates were performed; β-actin expression was used as an internal control. Treatment of AGS cells with euphol decreased TGF-β induced fibronectin expression in a dose dependent manner (lanes 7 through 12). At 40 μg/ml, euphol inhibited fibronectin expression by ~90% as compared with that in cells treated with TGF-β alone (lane 7 versus lane 11) in AGS cells.

## Discussion

Previous studies have suggested that direct interaction of triterpenoids with the plasma membrane might modulate membrane protein signaling by altering membrane structure [52–54]. Euphol is an alcohol tetracyclic triterpene with a wide range of pharmacological properties, including anti-cancer and anti-inflammatory activities [55]. The chemical structure of euphol is similar to that of cholesterol, an essential component of rafts. Because euphol and its derivatives are hydrophobic molecules that are structurally similar to cholesterol, we predicted they would incorporate into the plasma membrane, and more specifically into lipid rafts. Using surface plasmon resonance (SPR) analysis and liquid chromatography-tandem mass spectrometry, we demonstrated that euphol interacted with the model membrane (S4 Fig), and also preferentially intercalated into the lipid raft microdomains of the plasma membrane fractions (S5 Fig).

**Fig 8:**
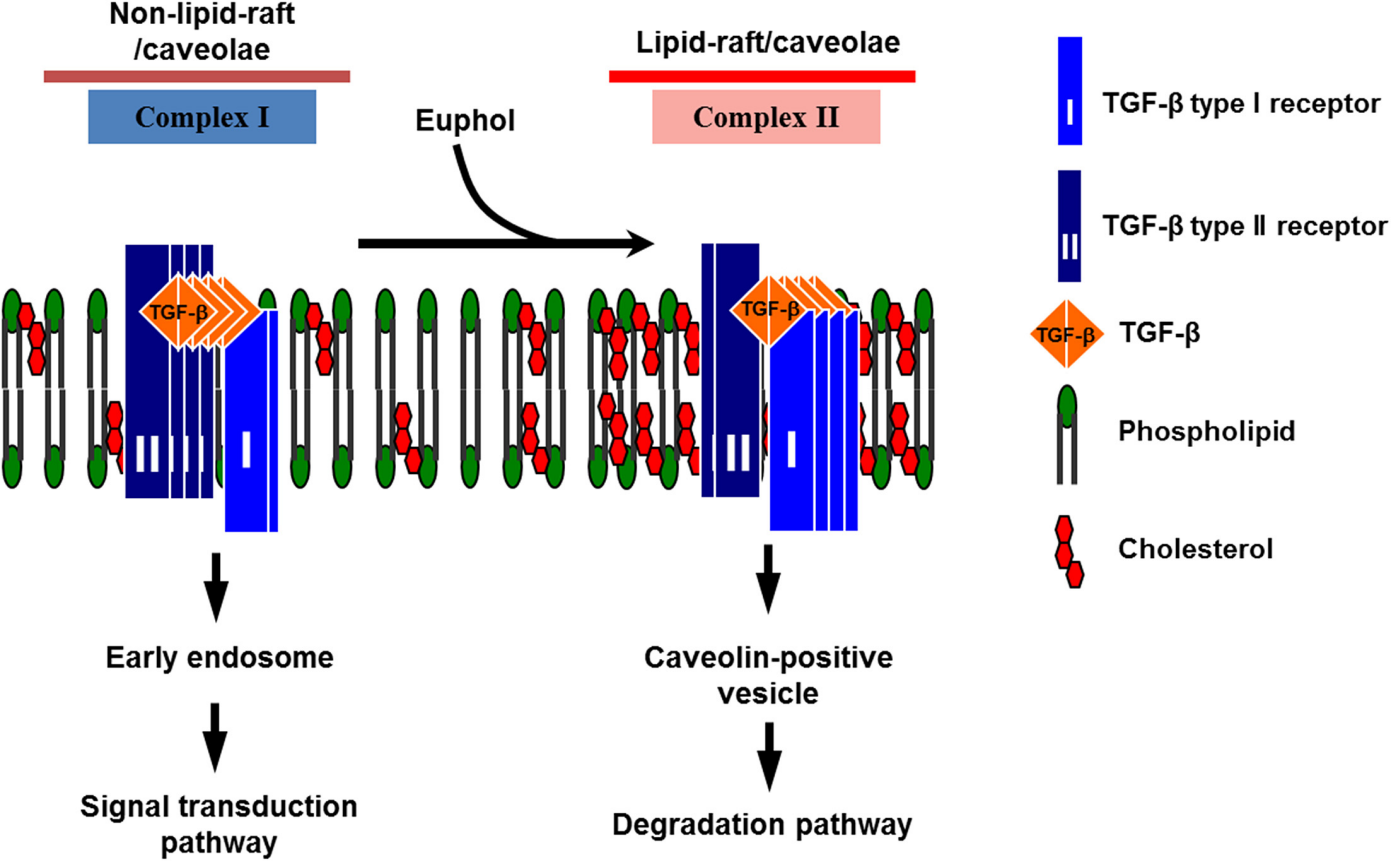
A model for the effect of euphol on TGF-β receptor partitioning between lipid rafts/caveolae- and clathrin-mediated endocytosis. In mammalian cells, there are 2 major TβR-I-TβR-II complexes (complex I and complex II) present on the cell surface. Complex I and complex II are mainly localized in the non-lipid raft and lipid raft/caveolae microdomains of the plasma membrane, respectively. Euphol increases the formation and/or stabilization of lipid rafts/caveolae by integration into the plasma membrane, thereby increasing the localization of TβR-I and TβR-II in lipid rafts/caveolae (as complex II), facilitating rapid degradation of TGF-β and attenuating Smad dependent-TGF-β signaling.

The core structure of the triterpene euphol contains 4 fused rings, designated A, B, C, and D. Ring C is fused to a 5-member D ring, in an arrangement identical to that of cholesterol. Both euphol and cholesterol have a hydroxyl group at C-25 and a planar structure [55, 56]. We speculate that euphol modulated TGF-β receptor activity because its stereo core structure is more similar to cholesterol than other triterpenes, such as the lupine-type triterpene betulinic acid. As a result of the similarities, euphol would tend to align with the cholesterol in lipid rafts. Theoretically, the hydroxyl group of euphol should compete with the hydroxyl group of cholesterol, owing to its hydrophilic nature. Because of its cholesterol-like core structure and hydroxyl group at C-25, euphol insertion either induces removal of cholesterol from lipid rafts or causes lipid raft membranes to expand [56]. Whichever the case, it perturbs the structure of the lipid raft, resulting in modulation of TGF-β receptor function. More specifically, euphol-induced expansion and stabilization of lipid raft domains stimulate recruitment of TGF-β receptors, accelerate TGF-β receptor degradation, and suppress TGF-β responsiveness (Fig 8).

We previously reported that suppressed TGF-β responsiveness in the aortic endothelium plays an important role in the pathogenesis of atherosclerosis in hypercholesterolemic animals [22, 23]. A high concentration of cholesterol in the culture medium suppresses TGF-β responsiveness in cultured cells, including endothelial cells, by causing accumulation of cell-surface TGF-β-TGF-β receptor complexes in lipid rafts/caveolae of the plasma membrane, facilitating rapid degradation of these complexes, and thus attenuating TGF-β-stimulated signaling and related responses [22–24]. This effect of cholesterol is believed to be mediated by increasing formation of, or stabilization of, lipid rafts/caveolae, presumably via direct integration of cholesterol into the plasma membranes of target cells [22, 23]. Lipid rafts/caveolae are thought to form platforms for the aggregation for proteins complexes involved in many key cellular functions, including signal transduction, membrane fusion, cytoskeleton organization, lipid sorting, protein trafficking, and localization and activity of specific membrane channels [25–28]. In this study, euphol treatment induced segregation of TGF-β receptors to lipid rafts in Mv1Lu cells, as well as in the AGS and MKN45 gastric cancer cell lines (Fig 4A, 4B and 4C).

Segregation of TGF-β receptors to lipid rafts suppressed canonical TGF-β-dependent signaling [22, 23, 38, 57]. Several lines of evidence presented herein indicate that euphol is an effective TGF-β antagonist that is capable of suppressing Smad2-dependent TGF-β signaling in various cell types: (1) euphol treatment inhibited the effects of TGF-β1 induced signaling, including Smad2 phosphorylation and nuclear translocation; (2) euphol treatment suppressed luciferase activity in TGF-β stimulated Mv1Lu cells expressing a luciferase reporter gene driven by the PAI-1, fibronectin, and collagen promoters; and (3) the effect of euphol on TGF-β signaling is rapid, reversible, and specific.

In consistent with previous reports [22, 23, 31, 58], we found that treatment of cells with nystatin also disrupts lipid rafts and enhances TGF-β-stimulated canonical signaling (Figs 4 and 3). Since nystatin sequesters cholesterol but does not deplete cellular cholesterol levels, the cholesterol content of nystatin-treated cells is not different from that of untreated cells [31, 59, 60]. Methyl-β-cyclodextrin (MβCD) depletes cholesterol and/or other sterols from plasma membranes and thus disrupts lipid rafts [61]. Although MβCD and nystatin disrupt lipid rafts by different mechanisms, they exhibited similar effects on TGF-β canonical signaling. Here we demonstrate that treatment of cells with either 0.5% MβCD or 25 μg/mL nystatin prior to TGF-β stimulation suppresses euphol’s ability to inhibit TGF-β signaling. Lipid rafts appear to play a regulatory role in TGF-β canonical signaling.

TGF-β promotes epithelial-mesenchymal transition (EMT) via the Smad pathway and up-regulates many genes, including PAI-1, fibronectin, and type I collagen [50, 51]. We believe euphol may regulate EMT by inhibiting TGF-β stimulated Smad2 phosphorylation and the subsequent nuclear translocation of Smad (Fig 3). Results of our study support this. Euphol treatment suppressed TGF-β regulation of PAI-1, fibronectin, and type I collagen (Fig 2). Furthermore, it also suppressed TGF-β-stimulated expression of fibronectin mRNA transcript and protein (Fig 7). The effect of euphol on EMT might be mediated through inhibition of Smad2 phosphorylation and subsequent nuclear translocation of Smad (Fig 3).

Transforming growth factor-β has dual roles in tumor initiation and growth [43]. In the early phase of progression, it inhibits of tumor epithelial cell growth by inducing cell cycle arrest and apoptosis. In later stages, the tumor promoting effects of TGF-β are unmasked, leading to migration, invasion, and metastasis.

The present study demonstrates that euphol suppresses TGF-β signaling characterized by Smad2 phosphorylation and PAI-1 transcription, and this effect is at least partially attributable to lipid-raft segregation and down-regulation of TGF-β receptors. Therefore, euphol has substantial potential as a treatment for cancer as well as fibrosis and other diseases.

## Supporting Information

S1 Fig Euphol treatment does not exhibit cytotoxic effects on Mv1Lu cells. Cells were treated with increasing concentrations of euphol (0, 10 20, 40, and 60 μg/ml) follow by MTT assay.
(TIF)

S2 Fig Mv1Lu cells in DMEM with 0.1% FCS were treated with increasing concentrations of euphol (0, 5, 10, 20, 40, and 60 μg/ml) for 1 hour and then stimulated with or without TGF-β for 30 min. Total protein extracts from treated cell were immunoblotted with anti-pSmad2, anti-Smad2/3, anti-TβR-II, anti-transferrin receptor, anti-caveolin-1 and anti-β-actin antibody. Protein expression of β-actin was used as a loading control for the same amount of cell lysates. Except pSmad2, the levels of other proteins were not changed during the short-term (total 1.5 hour) treatment.
(TIF)

S3 Fig Euphol treatment does not change mRNA transcript levels of TGF-β receptors. (A) AGS cells were treated with or without euphol (40 μg/ml) for 24 h. Total RNA was isolated and the expression of TβR-I and TβR-II was determined by RT-PCR. GAPDH was used as a loading control.
(TIF)

S4 Fig Surface plasmon resonance sensograms of the interactions of euphol with lipid coated-L1 sensor chip. The L1 chip allows for coating of intact lipid vesicles, and this L1 chip was used to test the binding of euphol on an artificial lipid membrane with a Biacore 3000. Euphol was injected at increasing concentrations (0, 25 and 50 μg/ml) at a flow rate of 30 μl/min to determine the response in signal (RU) at each respective euphol concentration.
(TIF)

S5 Fig Euphol preferentially insert into lipid-raft microdomains in Mv1Lu cells. Sucrose gradient fractions from cells treated with euphol for 4 hours were analyzed by liquid chromatography coupled with tandem mass spectrometry (LC/MS/MS). Liquid chromatography profile of extracted euphol from lipid-raft (red line) and non-lipid-raft (green line). The peak at 5.42 min corresponds to euphol.
(TIF)
