# Supplementary figures and images for: Euphol from Euphorbia tirucalli Negatively Modulates TGF-β Responsiveness via TGF-β Receptor Segregation inside Membrane Rafts
Source: PLoS One. 2015 Oct 8;10(10):e0140249. doi: 10.1371/journal.pone.0140249 (PMC4598150; doi:10.1371/journal.pone.0140249)

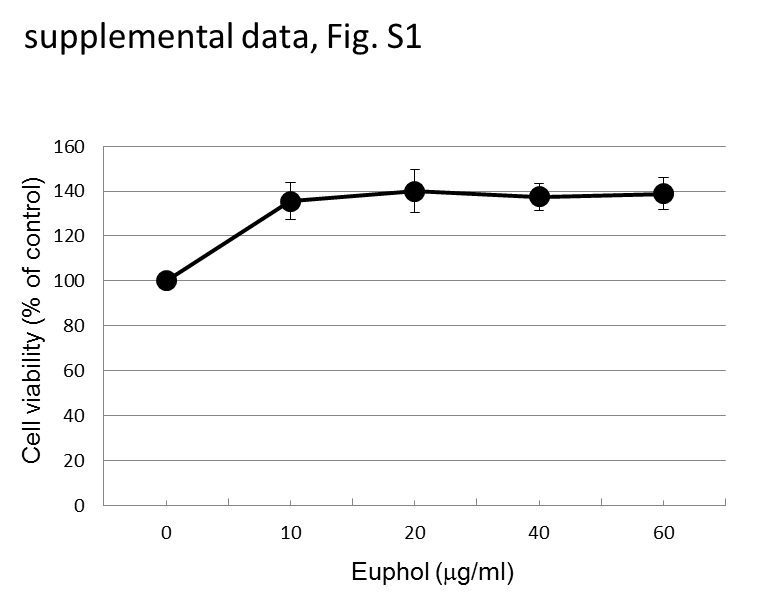

Supplement: S1 Fig — Cells were treated with increasing concentrations of euphol (0, 10 20, 40, and 60 μg/ml) follow by MTT assay. (TIF) [file pone.0140249.s001.tif]

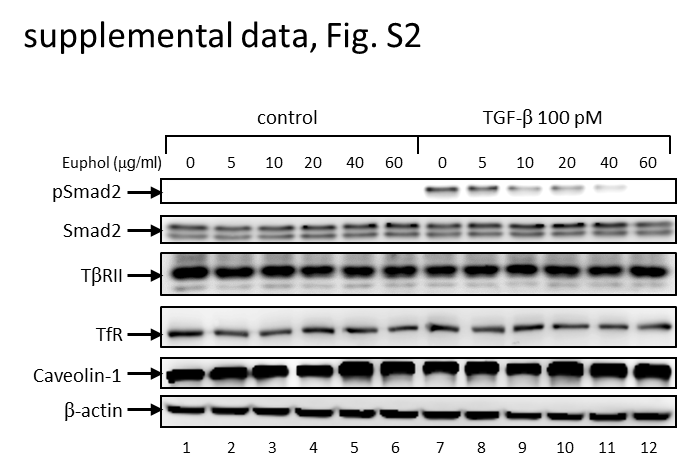

Supplement: S2 Fig — Total protein extracts from treated cell were immunoblotted with anti-pSmad2, anti-Smad2/3, anti-TβR-II, anti-transferrin receptor, anti-caveolin-1 and anti-β-actin antibody. Protein expression of β-actin was used as a loading control for the same amount of cell lysates. Except pSmad2, the levels of other proteins were not changed during the short-term (total 1.5 hour) treatment. (TIF) [file pone.0140249.s002.tif]

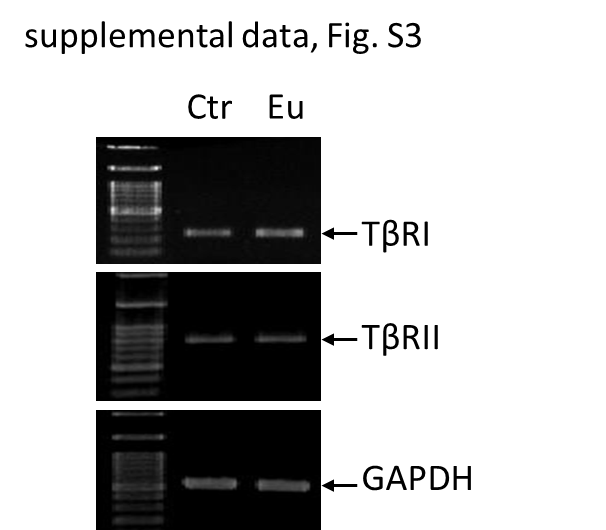

Supplement: S3 Fig — (A) AGS cells were treated with or without euphol (40 μg/ml) for 24 h. Total RNA was isolated and the expression of TβR-I and TβR-II was determined by RT-PCR. GAPDH was used as a loading control. (TIF) [file pone.0140249.s003.tif]

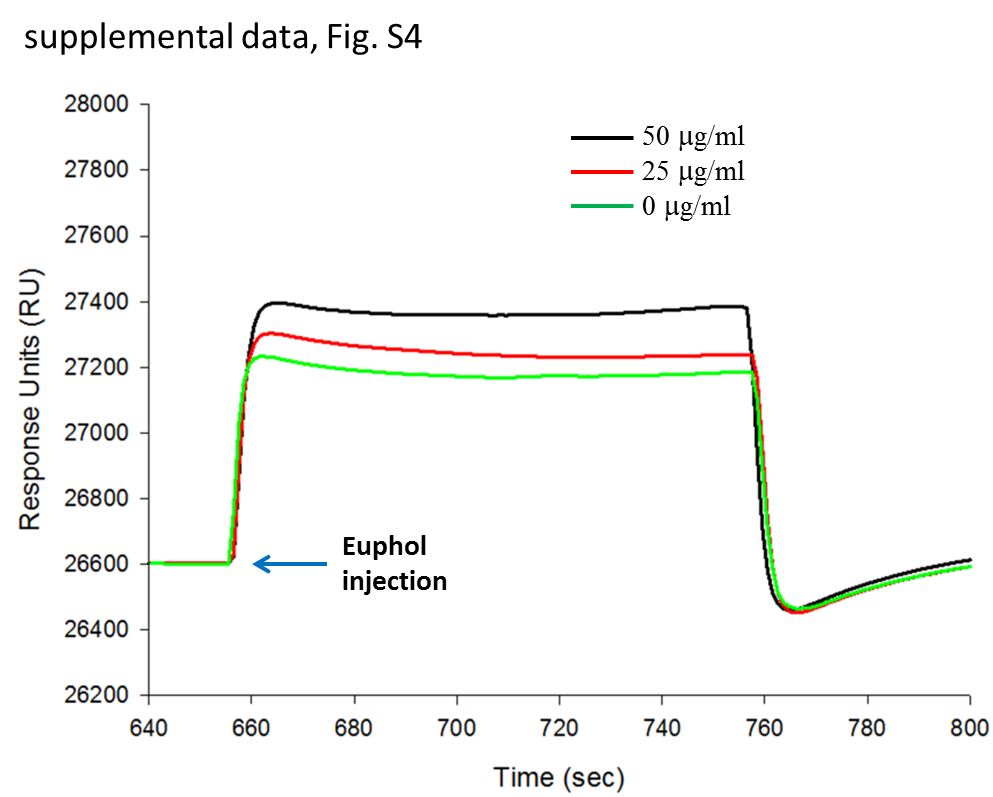

Supplement: S4 Fig — The L1 chip allows for coating of intact lipid vesicles, and this L1 chip was used to test the binding of euphol on an artificial lipid membrane with a Biacore 3000. Euphol was injected at increasing concentrations (0, 25 and 50 μg/ml) at a flow rate of 30 μl/min to determine the response in signal (RU) at each respective euphol concentration. (TIF) [file pone.0140249.s004.tif]

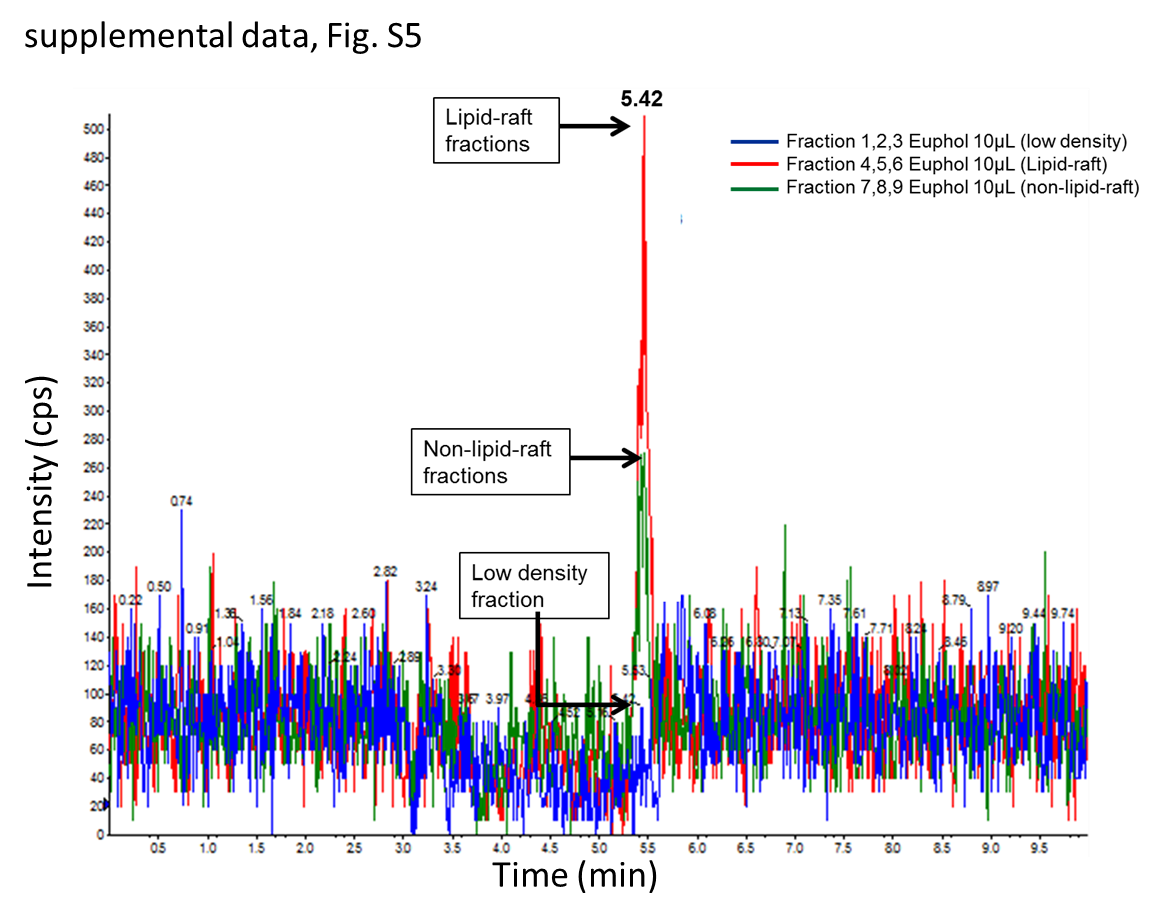

Supplement: S5 Fig — Sucrose gradient fractions from cells treated with euphol for 4 hours were analyzed by liquid chromatography coupled with tandem mass spectrometry (LC/MS/MS). Liquid chromatography profile of extracted euphol from lipid-raft (red line) and non-lipid-raft (green line). The peak at 5.42 min corresponds to euphol. (TIF) [file pone.0140249.s005.tif]
